# The illusion of functional stability: functional redundancy does not equal functional insurance in a natural-engineered aquatic continuum

**DOI:** 10.1093/ismeco/ycag229

**Published:** 2026-08-24

**Authors:** Qiong Tang, Zhe Li, Lunhui Lu

**Affiliations:** State Key Laboratory of Lake and Watershed Science for Water Security, Chongqing Institute of Green and Intelligent Technology, Chinese Academy of Sciences, Chongqing 400714, China; Three Gorges Laboratory, Chongqing 400714, China; Chongqing School, University of Chinese Academy of Sciences, Chongqing 400714, China; State Key Laboratory of Lake and Watershed Science for Water Security, Chongqing Institute of Green and Intelligent Technology, Chinese Academy of Sciences, Chongqing 400714, China; Three Gorges Laboratory, Chongqing 400714, China; Chongqing School, University of Chinese Academy of Sciences, Chongqing 400714, China; State Key Laboratory of Lake and Watershed Science for Water Security, Chongqing Institute of Green and Intelligent Technology, Chinese Academy of Sciences, Chongqing 400714, China; Three Gorges Laboratory, Chongqing 400714, China; Chongqing School, University of Chinese Academy of Sciences, Chongqing 400714, China

**Keywords:** functional redundancy, functional insurance, response diversity, ecosystem function

## Abstract

Functional redundancy—the capacity of multiple species to perform similar ecological functions—is widely regarded as a buffer that stabilizes ecosystem processes. However, whether functional redundancy truly translates into functional insurance remains unverified in microbial communities. Here, by integrating metagenomics and metatranscriptomics from 52 samples across a natural-engineered aquatic continuum in the Yanjin River watershed, China, results show that functional redundancy and functional insurance are fundamentally decoupled. We constructed a dual-layer analytical framework linking genomic functional overlap (effect trait) with transcriptional response heterogeneity (response trait) and developed a functional insurance index (FII) that quantifies whether redundant species respond differently to environmental variation. Our results reveal a striking paradox: engineered systems harbor the highest genomic functional redundancy yet the lowest functional insurance, because their functionally redundant species respond synchronously to perturbations—creating an illusion of stability. In contrast, natural communities exhibit lower absolute redundancy but significantly higher insurance, driven by diversified transcriptional responses among functionally similar species. Critically, only FII—not redundancy, response diversity, or asynchrony alone—significantly predicted CO_2_ and CH_4_ concentrations and DOM compositional stability, closing the evidence chain from genotype through phenotype to ecosystem function. These findings challenge the prevailing assumption that functional redundancy ensures functional insurance and reveal that conventional redundancy-based assessments may systematically overestimate or underestimate the functional stability of aquatic ecosystems.

## Introduction

Microbial communities are the principal drivers of biogeochemical cycling in aquatic ecosystems, mediating the transformation of carbon (C), nitrogen (N), phosphorus (P), and sulfur (S) across diverse environmental compartments [1, 2]. In small watersheds, microbial communities traverse steep environmental gradients—from the organic-rich, oxygen-depleted conditions within sewer pipes through the engineered selective pressures of wastewater treatment to the comparatively oligotrophic, hydrologically dynamic conditions of natural streams and reservoirs. How microbial communities maintain functional stability across such heterogeneous environments remains a central question in environmental microbiology, with direct implications for water quality management and greenhouse gas emission control [3–5].

Functional redundancy, the phenomenon whereby multiple species are capable of performing the same or similar ecological functions, has long been invoked as a key mechanism underpinning functional stability [6, 7]. The rationale is intuitive: if one species is lost, functionally equivalent species can compensate, thereby buffering ecosystem processes. In microbial ecology, functional redundancy has traditionally been inferred from the overlap of taxonomic or qualitative functional gene annotations [8, 9]. Recent advances have enabled more rigorous quantification: notably, Jiang *et al.* [10] proposed a hierarchical framework that moves the field from qualitative descriptions to quantitative, network-based assessments of functional redundancy structure. Yet, this framework considers only effect trait (i.e. which species can perform the same function), neglecting response trait (how species respond to environmental change) [11, 12]. This omission raises a fundamental but untested question: does high functional redundancy genuinely guarantee functional insurance, or in certain environments, could it create an illusion of functional stability?

The relationship between biodiversity and ecosystem stability has been a central concern in ecology for decades [13]. The insurance hypothesis provided a foundational mechanism, proposing that biodiversity stabilizes ecosystem processes because species respond differently to environmental fluctuations—so that when some species decline, others compensate [11, 12, 14–16]. Loreau and de Mazancourt [15] further formalized this idea, demonstrating mathematically that ecosystem stability depends not on species richness per se but on the asynchrony of species’ responses to perturbation. In parallel, the concept of response diversity—the range of responses to environmental change among species contributing to the same ecosystem function—was developed as the mechanistic bridge between redundancy and insurance [12, 17]. Despite these theoretical advances, practical application of these concepts to microbial systems has remained remarkably limited. Most studies of microbial functional redundancy have relied exclusively on genomic or taxonomic data to infer functional overlap [9, 18], implicitly assuming that genomic redundancy translates into functional insurance. However, this assumption conflates two conceptually distinct properties: the capacity for functional substitution (effect trait overlap) and the likelihood of compensatory response under stress (response trait diversity). Our dual-layer framework explicitly decouples these two dimensions for the first time in microbial ecology, integrating metagenomics-derived effect traits with metatranscriptomics-derived response traits to test whether redundancy genuinely provides insurance—or merely creates the illusion of stability.

**Figure 1:**
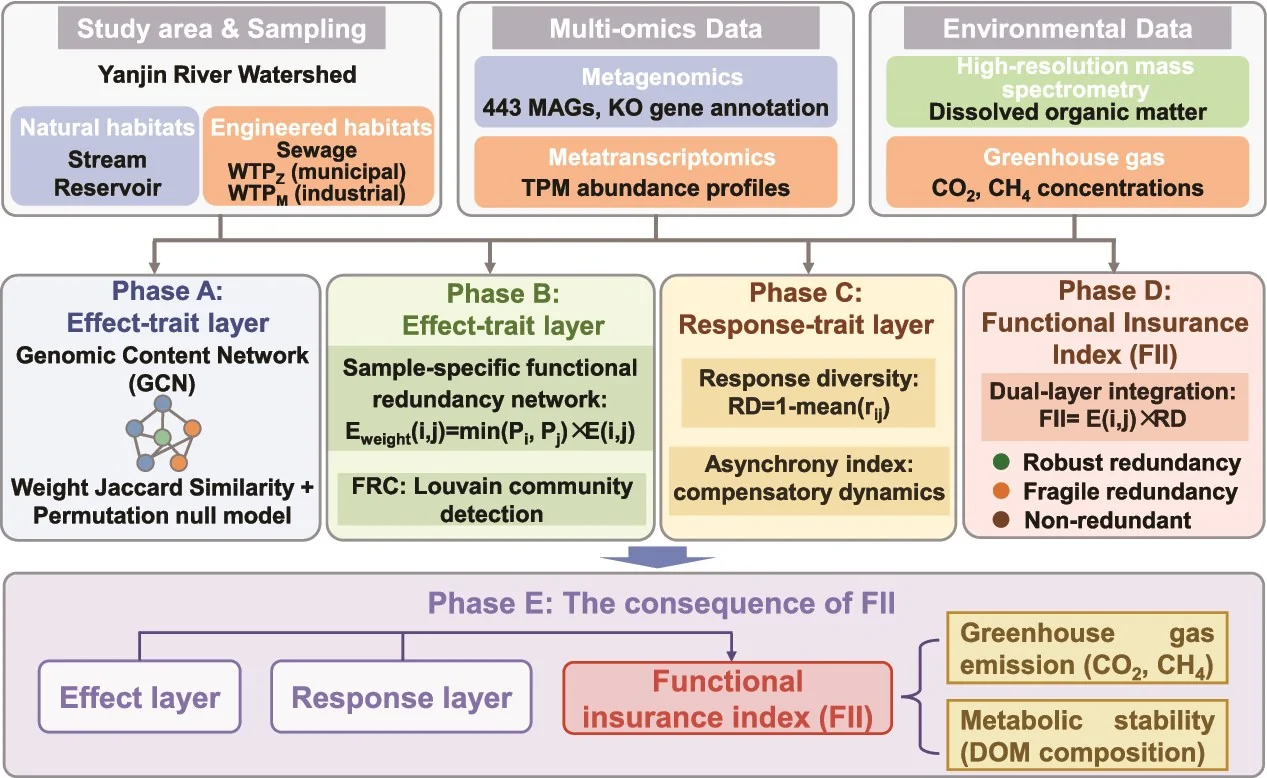
Conceptual framework for assessing microbial functional insurance through dual-layer trait integration. Samples were collected from the Yanjin River watershed across natural habitats (streams, reservoirs) and engineered habitats (sewage pipelines, municipal wastewater treatment plant WTP_Z_, industrial wastewater treatment plant WTP_M_). Metagenomic sequencing yielded 443 MAGs with KEGG Orthology (KO) gene annotations, complemented by metatranscriptomic TPM abundance profiles. Environmental parameters included DOM composition characterized by high-resolution mass spectrometry and greenhouse gas (CO_2_, CH_4_) concentrations. The analytical framework comprises five phases: Phase A constructs a GCN by quantifying pairwise effect trait similarity using WJS, with a statistical significance threshold determined by a permutation null model (*n* = 1000). Phase B generates sample-specific FRN, where edge weights incorporate local species abundances, and applies Louvain community detection to delineate FRCs. Phase C operates at the response trait layer, quantifying response diversity within each FRC [RD = 1 − mean(r_ij_)], where r_ij_ is the Pearson correlation coefficient of abundance dynamics between species pairs, alongside an asynchrony index capturing compensatory dynamics. Phase D integrates both trait layers into an FII, classifying each FRC into three categories: robust redundancy (high effect similarity + high response diversity), fragile redundancy (high effect similarity + low response diversity), and non-redundant (low effect similarity). Phase E evaluates the ecological consequences of FII by examining its relationship with greenhouse gas (CO_2_, CH_4_) and metabolic stability (DOM composition).

Therefore, we collected samples across the Yanjin River small watershed in Guizhou, China, encompassing natural-to-engineered aquatic continuum. By integrating metagenomic data to characterize effect trait (genomic functional similarity) and metatranscriptomic data to characterize response trait (transcriptional response heterogeneity), we developed an “effect-response” dual-layer functional redundancy framework and proposed the functional insurance index (FII) as a novel composite metric that quantitatively distinguishes robust from fragile functional redundancy. We tested three hypotheses: (H1) if functional redundancy is governed primarily by effect trait overlap, whereas functional insurance additionally requires response trait diversity, then these two properties should be decoupled along environmental gradients—i.e. habitats with high redundancy may not necessarily possess high insurance, and vice versa; (H2) if environmental homogenization in engineered systems reduces the heterogeneity of selective pressures, then response diversity among functionally redundant taxa should be lower in engineered than in natural systems—because strong environmental filtering narrows niche differentiation; and (H3) if the insurance hypothesis applies to microbial communities, then a metric integrating both effect trait and response trait (i.e. FII) should predict ecosystem functional stability more effectively than redundancy or response diversity alone—because insurance requires both components simultaneously. The conceptual framework and study design integrating these analytical phases were illustrated in Fig. 1.

## Materials and methods

### Study area and sampling design

The Yanjin River Basin, a first-class tributary of the Chishui River in China’s “Wine Capital” Renhuai City, Guizhou Province, spans 38 km with an average annual flow of 4.35 m^3^/s. A total of 52 samples were collected from 26 sites across two seasons (May 2023 and January 2024), covering three habitat categories (Fig. S1): (i) natural water bodies including reservoirs (R1 ~ R3) and streams (S1 ~ S10); (ii) urban sewage pipelines receiving discharge from a hospital, market, residential area, school, and samples before the wastewater treatment plant (P1 ~ P7); and (iii) the inlet, aerobic tank, outlet of municipal wastewater treatment plant WTP_Z_ (W_Zi_, W_Za_, W_Zo_); industrial wastewater treatment plant WTP_M_ (W_Mi_, W_Ma_, W_Mo_) samples.

At each site, water samples were collected for dissolved gas analysis (CO_2_, CH_4_) and dissolved organic matter (DOM) characterization using high-resolution mass spectrometry (details in Supplementary Method 1), while for microbial analysis, samples were filtered through 0.22-μm Millipore filters (Milford, MA, USA) and stored at −80°C for DNA/RNA extraction. Remaining samples were treated with H_2_SO_4_ to inhibit microbial activity prior to physicochemical analysis (Supplementary Method 2, Fig. S2).

### Metagenomic sequencing

Total genomic DNA was extracted using the MagAttract PowerSoil Pro DNA Kit (Qiagen) and quantified via NanoDrop 2000 and TBS-380. Metagenomic libraries with 350-bp inserts were prepared through Covaris M220 fragmentation and the NEXTFLEX Rapid DNA-Seq Kit (Bioo Scientific). Sequencing was performed on the Illumina NovaSeq 6000 platform (2 × 150 bp; Majorbio, China).

Raw reads were quality-filtered using fastp (v0.23.0) [19]. The remaining high-quality reads were assembled into contigs using MEGAHIT (v1.1.2) [20], based on succinct de Bruijn graphs. Contigs ≥300 bp were retained for downstream analysis. Open reading frames (ORFs) were predicted using Prodigal (v2.6.3) [21], and a non-redundant gene catalog was constructed using CD-HIT (v4.6.1) with 90% sequence identity and 90% coverage [22]. To calculate gene abundance, high-quality reads were aligned to this catalog using SOAPaligner (v2.21) [23] with a 95% identity threshold.

Taxonomic assignment of the non-redundant genes was performed using DIAMOND (v2.0.13) [24] against the NCBI NR database (e-value cutoff of 1 × 10^−5^). Functional annotation was conducted using KOfam against the KEGG database, with a specific focus on key genes involved in C, N, P, and S cycling. To recover individual microbial genomes, metagenome-assembled genomes (MAGs) were reconstructed using MetaBAT2 [25] and quality-assessed via CheckM2 [26]. A total of 442 medium-to-high quality MAGs (completeness ≥50% and contamination <10%) were retained for subsequent analyses.

### Metatranscriptomic sequencing

Total RNA was extracted from samples using the Soil RNA Kit (Majorbio, China) according to the manufacturer’s protocols. To concentrate the functional information, ribosomal RNA (rRNA) was depleted using the RiboCop rRNA Depletion Kit (Lexogen, Austria). The remaining mRNA was then used to construct sequencing libraries and sequenced on the Illumina platform (as described above).

Raw metatranscriptomic reads were processed for quality control using fastp (v0.19.6) [19] to remove low-quality reads and adapters. Residual rRNA sequences were further identified and eliminated using SortMeRNA (v2.1b) [27] against multiple rRNA databases. To quantify the transcriptional activity of specific microbial populations, the high-quality mRNA reads were mapped to a non-redundant gene catalog predicted from the recovered MAGs. This mapping and quantification process was performed using RSEM (v1.3.3) (integrated with Bowtie2).

Gene expression levels were normalized as transcripts per million (TPM) to account for variations in sequencing depth and gene length. The overall transcriptional activity of each MAG was then determined by aggregating the TPM values of all constituent genes assigned to that specific genome. For functional and taxonomic characterization, the representative sequences of the gene catalog were aligned against the NCBI NR database using DIAMOND (v0.8.35) [24] with an e-value cutoff of 1 × 10^−5^. Functional annotations were further assigned based on the KEGG database to evaluate the metabolic potential and active pathways.

### Effect-trait layer: functional redundancy hierarchy construction

#### Genomic content network construction

To quantify pairwise functional similarity among MAGs *E(i, j)*, we computed the weighted Jaccard similarity (WJS) based on a species–functional gene matrix B, where B_ik_ represents the copy number of the *k*-th KO gene (related to C, N, P, or S cycling) in MAG i. This matrix (442 MAGs × C/N/P/S-related KO genes) encodes the effect-trait dimension (genomic functional similarity) of each MAG. Unlike the binary Jaccard index, WJS accounts for variation in gene copy number, providing a more nuanced measure of functional overlap.


(1) \begin{equation*} E\left(i,j\right)=\frac{\sum_k\min \left({B}_{ik},{B}_{jk}\right)}{\sum_k\max \left({B}_{ik},{B}_{jk}\right)} \end{equation*}

To exclude spurious similarities arising from universally shared genes, we constructed a permutation null model based on 1000 random shuffles of the gene matrix. The observed similarity distribution was significantly distinct from the randomized null distribution (*P* < .05), and the 95th percentile of the null distribution (threshold = 0.2456) was adopted as the significance cutoff (Fig. S3). Only species pairs exceeding this threshold were retained for subsequent network construction, ensuring that the identified functional redundancy was statistically robust and reflected genuine functional overlap.

#### Personalized functional redundancy network

For each sample, we constructed a personalized functional redundancy network (FRN) by weighting each GCN edge [10] with the sample-specific relative abundances of the connected MAGs:


(2) \begin{equation*} {E}_{Weight}{\left(i,j\right)}^{(s)}=\min \left({P}_i^{(s)},{P}_j^{(s)}\right)\times E\left(i,j\right) \end{equation*}

where P*_i_*^(s)^ is the relative abundance of MAG i in sample s. The application of min (P_i_, P_j_) is grounded in the principle that the capacity for functional substitution is constrained by the less abundant partner, reflecting the limiting nature of redundancy [28]. Only MAG pairs with both species present (abundance >0) in the focal sample were included.

Total functional redundancy (TFR) was computed as the sum of all edge weights:


(3) \begin{equation*} {TFR}^{(s)}=\sum_{\left(i,j\right)\in E}{E}_{Weight}{\left(i,j\right)}^{(s)} \end{equation*}

Normalized functional redundancy was calculated as TFR divided by the maximum possible redundancy (assuming WJS = 1 for all connected pairs).

#### Functional redundancy cluster detection

Each sample-specific FRN was partitioned into functional redundancy clusters (FRCs) using the Louvain algorithm for modularity maximization [29], as implemented in NetworkX. Edge weights were incorporated during community detection. Isolated nodes (degree = 0) were excluded from the partitioning. Modularity was calculated for each partition to assess the quality of community structure.

### Response-trait layer: response diversity and asynchrony index quantification

#### Response diversity

For each identified FRC, response diversity was quantified using the metatranscriptomic expression profiles of all MAGs belonging to that cluster:


(4) \begin{equation*} {RD}_l=1-\overline{r_l}=1-\frac{1}{\mid{T}_l\mid}\sum_{\left(i,j\right)\in{T}_l} Cor\left(i,j\right) \end{equation*}

(5) \begin{equation*} RD=\frac{\sum_{l=1}^n{RD}_l}{\mid{FRC}_l\mid } \end{equation*}

RD*_l_* represents the response diversity of the *l*-th FRC; $\overline{r_l}$ denotes the average pairwise correlation among all members within this FRC; T*_l_* represents the set of all possible MAGs pairs within the *l*-th FRC; ∣T*_l_*∣ means the total number of MAGs pairs in this set; Cor(i, j) represents the Pearson correlation coefficient between the expression levels of MAG i and MAG j in this sample; |FRC*_l_*| indicates the number of FRCs in this sample. The higher the response diversity, the greater the differences in environmental response among the species within this cluster.

#### Asynchrony index

The asynchrony index quantifies the degree of compensatory dynamics within each FRC [30]:


(6) \begin{equation*} {Asy}_l=1-\frac{Var\left(\sum{T}_i\right)}{{\left(\sum \sqrt{Var\left({T}_i\right)}\right)}^2} \end{equation*}

(7) \begin{align*} Asy=\frac{\sum_{l=1}^n{Asyn}_l}{\mid{FRC}_l\mid } \end{align*}

As y*_l_* represents the asynchrony index of the *l*-th FRC; T*_i_* is the expression levels of MAG i in the *l*-th FRC; Var(∑T*_i_*) means variance of the total expression levels of all MAGs within this cluster; $\sum \sqrt{Var\left({T}_i\right)}$ is sum of the standard deviations of each MAG within the cluster. Asynchrony index approaches 1 when species fluctuations are perfectly compensatory (one species increases as another decreases) and 0 when species fluctuate in perfect synchrony.

### Dual-layer integration: functional insurance index

#### FII quantification

FII integrates the effect-trait layer (genomic functional similarity) with the response-trait layer (transcriptional response heterogeneity):


(8) \begin{equation*} {FII}_l=\overline{E}\left(i,j\in{FRC}_l\right)\times{RD}_l \end{equation*}

(9) \begin{equation*} FII=\frac{\sum_{l=1}^n{FII}_l}{\mid{FRC}_l\mid } \end{equation*}

where $\overline{E}$ is the mean pairwise WJS among all MAG pairs within FRC*_l_*, computed from the full GCN similarity matrix. The definition of other indicators was the same as described above. This ensures that FII captures both the genomic potential for substitution and realized response heterogeneity.

#### FRC ecological classification

Each FRC was classified into three ecological types based on bimodal thresholding: (i) robust redundancy (functional similarity ≥0.30; response diversity ≥0.50), indicating functionally interchangeable species with diverse, compensatory environmental responses; (ii) fragile redundancy (functional similarity ≥0.30; response diversity <0.50), suggesting functionally similar species that nonetheless respond synchronously to perturbations; and (iii) non-redundant (unique function) (functional similarity <0.30), indicating FRCs lacking substantive functional overlap regardless of response patterns. The rationale for threshold selection was provided in Supplementary Method 3, Fig. S3.

### Statistical analysis

All statistical analyses were performed in Python (v3.11.9) using scipy.stats, pandas, and numpy. Network topology metrics (density, degree distribution, clustering coefficient, etc.) were computed using the NetworkX library in Python (v3.11.9). Hierarchical clustering of microbial and physicochemical profiles was aligned using dendextend package in R v4.1.1 [31]. Structural equation modeling (SEM) was conducted using the piecewiseSEM package in R v4.1.1, with model fit evaluated by Fisher’s C values (*P* > .05 indicating adequate fit). DOM compositional stability was analyzed by dividing samples into high-FII and low-FII groups (median = 0.366). Bray–Curtis distance-based principal coordinates analysis (PCoA) visualized patterns [32], while PERMANOVA (adonis, 999 permutations) and PERMDISP (betadisper) tested for differences in composition and dispersion, respectively. Differential expression analysis of carbon-metabolism genes between high-FII and low-FII groups was performed using edgeR with a false discovery rate (FDR) threshold of <0.05 and a fold-change cut-off of |log_2_FC| > 1.

## Results

### The metacommunity genomic content network reveals extensive functional overlap across the sewage–river continuum

To quantify the functional redundancy among microorganisms, we calculated pairwise WJS for all MAGs based on the species–functional gene matrix encompassing key C, N, P, and S cycling genes. Then, a weighted genomic content network (GCN) comprising 442 nodes (MAGs) and 17 107 edges was constructed (Fig. S4a). Only two MAGs were completely isolated (degree = 0), indicating that functional overlap was nearly ubiquitous across the recovered genomes. The network exhibited a density of 0.176 and a mean degree of 77.4 ± 2.09 (median = 70), with edge weights ranging from 0.246 to 0.814 (mean = 0.307, median = 0.284). The average clustering coefficient was 0.628 ± 0.008, showing a pronounced modular and cohesive architecture within the functional space.

Taxonomically, the GCN was dominated by Pseudomonadota (136 MAGs, 30.8%), Bacteroidota (92 MAGs, 20.8%), Bacillota (68 MAGs, 15.4%), and Actinomycetota (57 MAGs, 12.9%), which collectively accounted for 79.9% of all nodes (Fig. S4a, b). Hub analysis revealed that the top three nodes ranked by weighted degree were MAG321 (*Alphaproteobacteria* class), MAG167 (*Bacteroidia* class), and MAG109 (*Gammaproteobacteria* class). Notably, among the top 20 hub nodes, 15 belonged to Pseudomonadota, suggesting that members of this phylum serve as central functional generalists with extensive genomic overlap across diverse metabolic pathways. The cross-phylum hub composition (Pseudomonadota, Bacteroidota, Actinomycetota, Verrucomicrobiota, and Acidobacteriota) indicated that functionally central positions in the GCN were not phylogenetically restricted, consistent with the ecological notion that functional redundancy transcends taxonomic boundaries. Overall, the topology of GCN demonstrated that at the metacommunity level, the GCN spanning the entire natural-engineered aquatic continuum harbors extensive functional overlap. This genomic-level redundancy architecture provides the structural foundation upon which personalized, sample-specific functional redundancy can subsequently be assessed.

### Engineered systems harbor the highest functional redundancy but distinct modular architectures

To further characterize the spatiotemporal heterogeneity of microbial functional redundancy, we constructed personalized FRNs for each of the 52 samples by weighting the GCN edges with sample-specific species abundances. In each sample-level FRN, an edge weight reflects not only genomic functional overlap but also the co-occurrence intensity among species in the sample, thereby translating a universal similarity metric into a context-dependent functional redundancy estimate.

**Figure 2:**
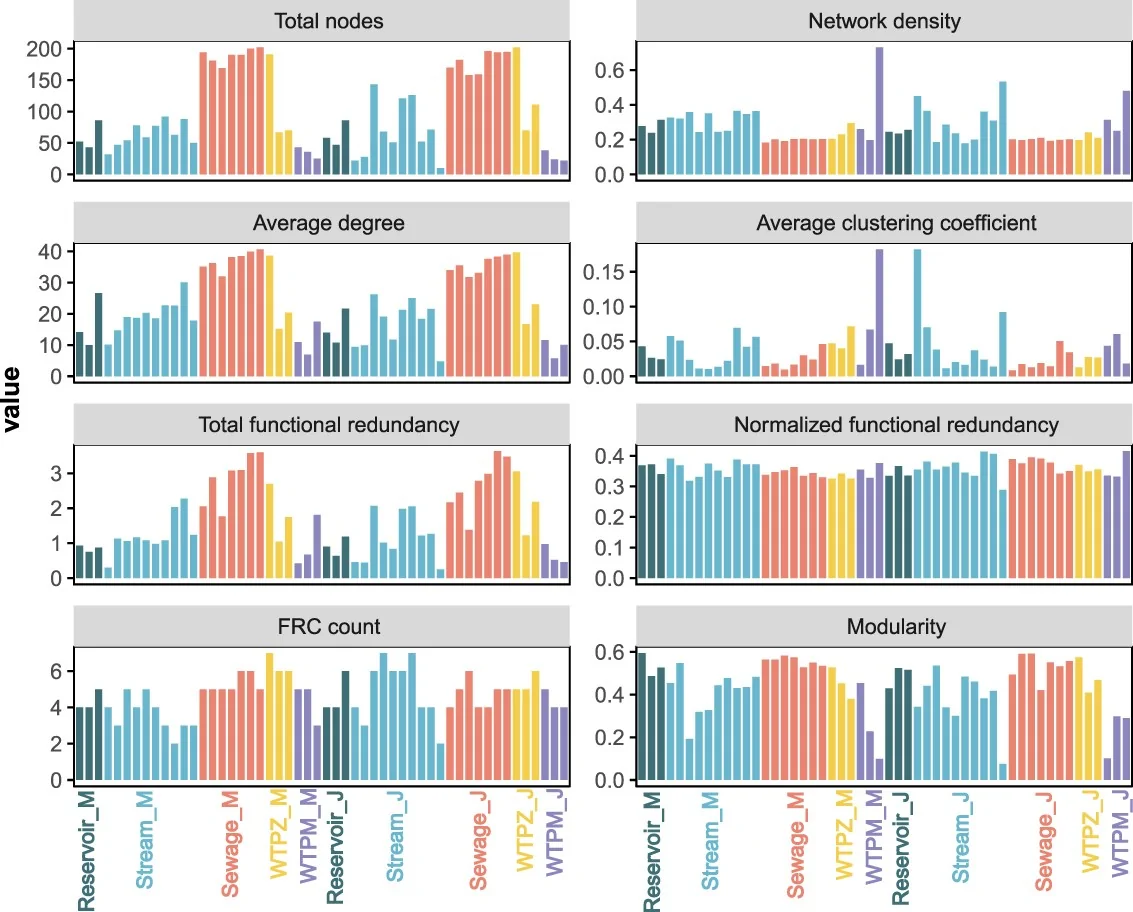
The topological parameters of personalized FRNs of various samples in each system (reservoir, stream, sewage, WTP_Z_, and WTP_M_) in May (M) and January (J).

Quantitative analysis of the resulting FRN topologies revealed substantial structural differentiation across samples (Fig. 2). Across all networks, the number of nodes ranged from 10 to 202 (mean = 99.7 ± 8.86), network density varied between 0.18 and 0.73 (mean = 0.27 ± 0.01), and the average degree spanned from 4.80 to 40.69 (mean = 22.8 ± 1.51). Notably, network density and average degree exhibited highly concordant fluctuation patterns, reflecting the tight coupling between local connectivity and global network compactness.

Habitat-level comparisons revealed clear hierarchical patterns in functional redundancy (Fig. 2). Sewage samples harbored the greatest species richness (184.6 ± 3.98 MAGs) and the highest TFR (TFR = 2.78 ± 0.19, Fig. S5), followed by the WTP_Z_ (119.5 ± 25.6 MAGs, TFR = 1.99 ± 0.33). In contrast, reservoir and stream samples exhibited markedly lower index (62.8 ± 8.37 MAGs, TFR = 0.88 ± 0.08; 68.0 ± 7.84 MAGs, TFR = 1.20 ± 0.14), while the WTP_M_ showed the lowest values (32.7 ± 3.73 MAGs, TFR = 0.81 ± 0.22). Generally, engineered systems (including sewage and WTP_Z_) tend to exhibit high functional redundancy, whereas WTP_M_ may not follow this pattern. This exception likely stems from the fact that WTP_M_ (industrial wastewater treatment) contains complex organic compounds, high concentrations of toxic substances, and extreme physicochemical conditions, etc., which impose strong selective pressures that narrow the functional repertoire and limit redundancy. Despite these large differences in TFR, normalized functional redundancy (adjusted for species pool size) remained remarkably stable across all habitats (Fig. S5c; overall mean = 0.357 ± 0.004).

Louvain-based community detection on each FRN partitioned the functional space into two to seven FRCs per sample. Modularity scores averaged 0.440 ± 0.018 across all samples but varied considerably among habitats: sewage networks exhibited the highest modularity (0.545 ± 0.012), followed by reservoir, stream, and WTP_Z_, whereas the WTP_M_ showed considerably lower modularity (0.245 ± 0.055). This pattern indicates that resource-rich sewer environments support well-defined functional modules, whereas the intense selective pressures in treated WTP effluents erode modular boundaries. Network modularity was positively correlated with TFR (Fig. S6; *P* < .001) and negatively correlated with network density (*P* < .001), indicating that higher modularity—reflecting tighter within-cluster functional overlap—is associated with greater redundancy and more differentiated inter-cluster boundaries. Seasonal comparison between January and May revealed no significant overall shift in TFR (Fig. S5d), suggesting that the genomic backbone of functional redundancy is relatively resilient to seasonal changes, although individual samples exhibited inter-seasonal variation.

### Response diversity declines systematically along the natural-to-engineered gradient

To assess whether functional redundancy confers genuine functional insurance, we quantified the response diversity and asynchrony index within each FRC using metatranscriptomic data. Response diversity measures the average dissimilarity in transcriptional profiles among functionally redundant species, while the asynchrony index captures the degree to which species within an FRC exhibit compensatory expression dynamics—i.e. when some species downregulate, others upregulate.

**Figure 3:**
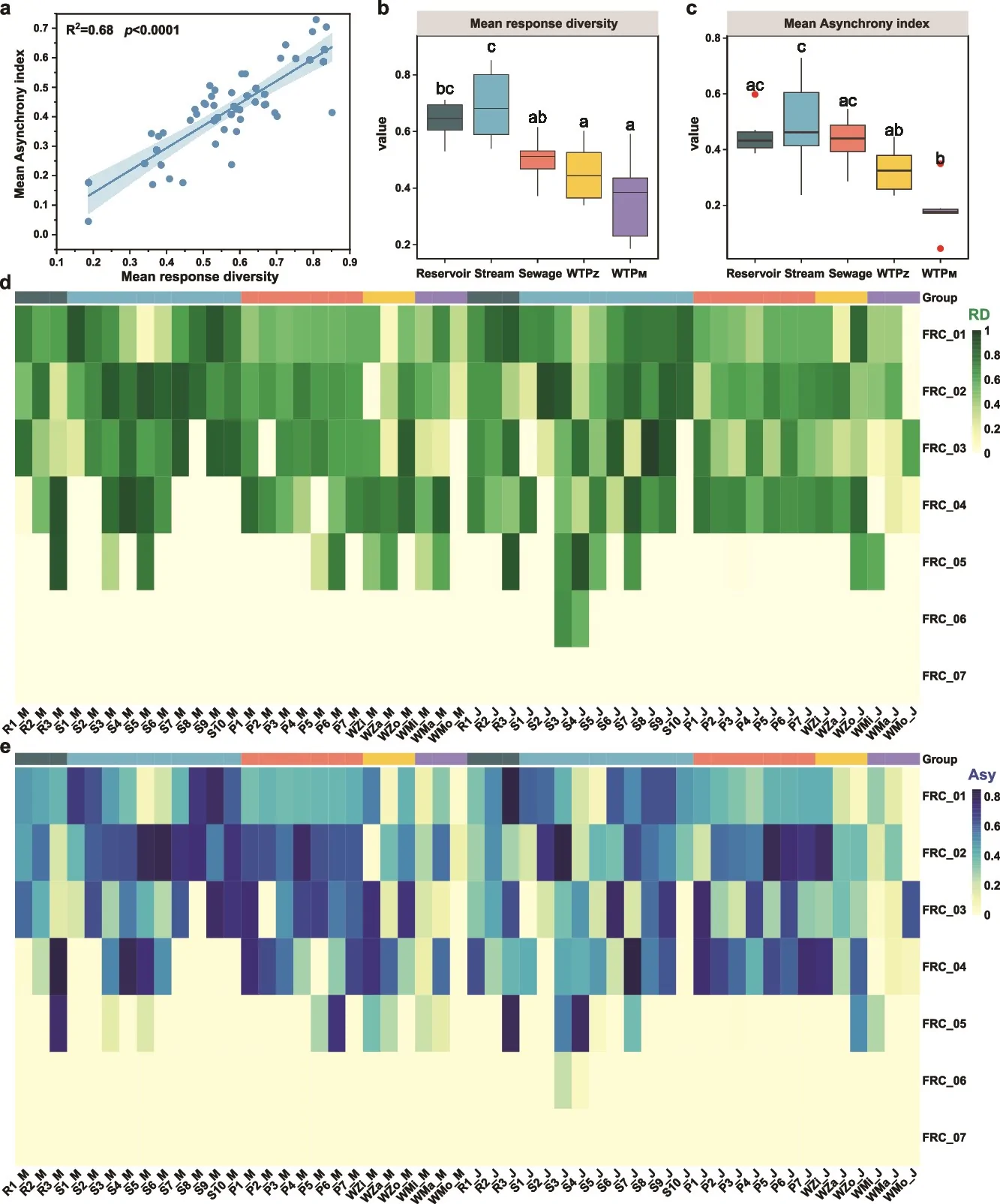
The changes in the response diversity and asynchrony index of each sample. (a) The liner relationship between mean response diversity and mean asynchrony index. Boxplot of mean response diversity (b) and asynchrony index (c) among reservoir, stream, sewage, WTP_Z_, and WTP_M_ systems; lowercase letters above the boxes represent the results of statistical significance test (*P* < .05, ANOVA with post hoc Tukey HSD test) for each group. Groups with different letters indicate significant differences between groups, and groups with the same letter means non-significant differences between groups. (d, e) The heatmap shows the response diversity (RD) and asynchrony index (Asy) of each FRC in each sample in May (M) and January (J).

Across all samples, the mean response diversity was 0.553 ± 0.018, and the mean asynchrony was 0.411 ± 0.017. A strong positive correlation was observed between these two metrics (Fig. 3a), indicating that FRCs with more divergent transcriptional responses also exhibited stronger compensatory dynamics—a prerequisite for the insurance effect.

Habitat-level comparisons revealed a pronounced gradient in both response diversity and asynchrony index from natural (reservoir, stream) to engineered environments (sewage, WTP_Z_, WTP_M_) (Fig. 3b and c). Stream samples exhibited the highest response diversity, followed by reservoir, sewage, and two WTPs systems. Aggregating natural versus engineered habitats revealed highly significant differences (response diversity: 0.685 ± 0.020 vs. 0.457 ± 0.023, *P* < .0001; asynchrony index: 0.494 ± 0.024 vs. 0.353 ± 0.026, *P* = .001). Within the WTP_M_ (industrial WTP), the effluent site (W_Mo_) showed the lowest response diversity and asynchrony index of any site in the entire dataset (Fig. 3d and e), substantially lower than the influent (W_Mi_) and mid-treatment stage (W_Ma_). This progressive decline along the treatment gradient indicates that the intense selective pressure of industrial WTP increasingly homogenizes microbial transcriptional responses, producing a community in which functionally redundant species respond synchronously to environmental perturbations rather than compensating for one another. The effluent site was not only far below all natural systems but also significantly lower than the WTP_Z_ (municipal WTP; *P* < .05), indicating that industrial treatment processes are particularly detrimental to the response asynchronously that underpins functional insurance.

These results demonstrated that while genomic-level functional redundancy is widespread at the continuum scale, its ecological insurance varies dramatically across habitats. Natural environments maintain high response diversity, enabling a genuine insurance effect. Conversely, the homogenizing selective pressures probably erode the response asynchronously within FRCs, rendering the remaining redundancy functionally “fragile.” This pattern was quantitatively captured in the subsequent analysis.

### Functional insurance decouples from redundancy

To integrate the effect-trait layer (genomic functional similarity) with the response-trait layer (response diversity), we computed the FII for each FRC across all samples. Overall, FII ranged from 0.0001 to 0.474 (mean = 0.176 ± 0.006). A bimodal thresholding approach—grounded in the permutation null model baseline and the mathematical logic of the insurance hypothesis—was applied to classify each FRC into one of three core ecological types: (i) robust redundancy, (ii) fragile redundancy, and (iii) non-redundant. Robust redundancy are clusters of species that share high genomic functional overlap and exhibit high transcriptional response diversity: these represent genuine insurance—the redundant species not only can perform the same function but respond differently to environmental perturbation, enabling compensatory dynamics. Fragile redundancy are clusters with high effect similarity but low response diversity: these represent the illusion of insurance—species are genomically redundant but respond synchronously to stress, meaning backups fail simultaneously. Non-redundant are clusters with low effect similarity regardless of response diversity, representing inherent vulnerability. The critical distinction between robust (genuine insurance) and fragile (illusion of insurance) redundancy FRCs has been illustrated graphically (Fig. S7).

**Figure 4:**
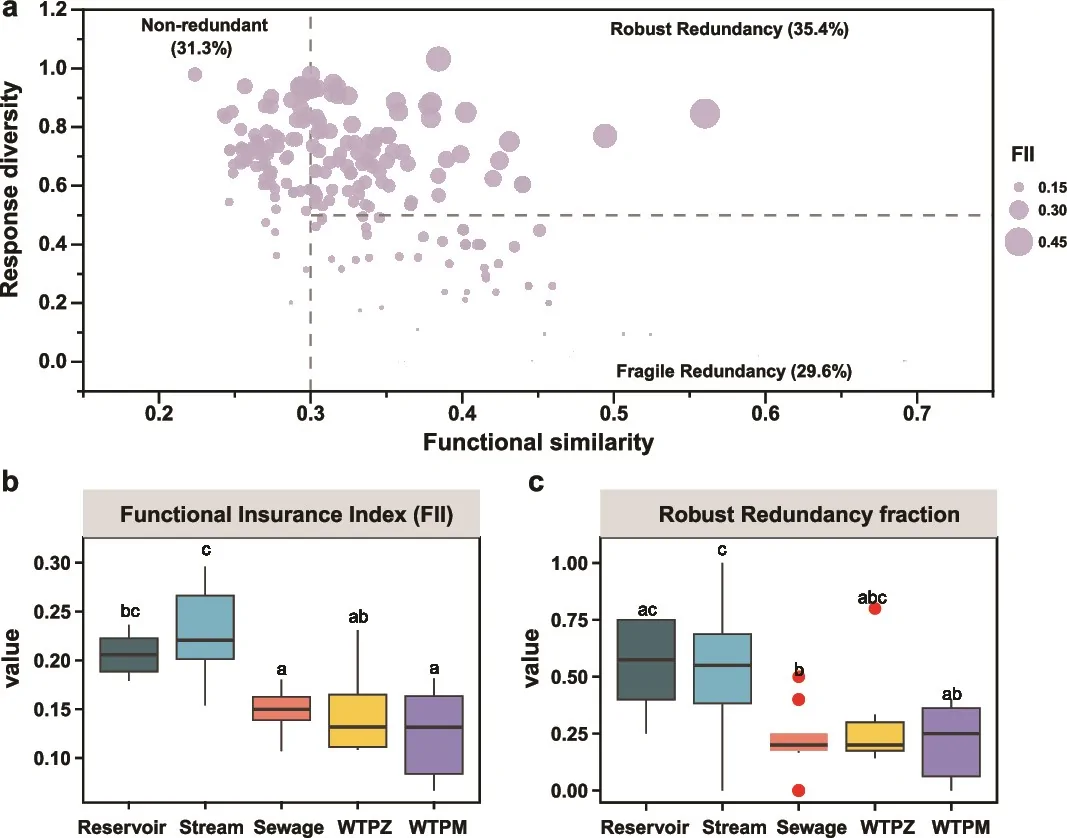
The characteristics of FII. (a) The proportion of different types of functional redundancy. The boxplot of functional insurance index (b) and robust redundancy fraction (c) in each system (reservoir, stream, sewage, WTP_Z_, WTP_M_). Lowercase letters above the boxes represent the results of statistical significance test (*P* < .05, ANOVA with post hoc Tukey HSD test) for each group. Groups with different letters indicate significant differences between groups, and groups with the same letter means non-significant differences between groups.

Among the 234 FRCs, 86 (35.4%) were classified as robust redundancy, 72 (29.6%) as fragile redundancy, and 76 (31.3%) as non-redundant (Fig. 4a). Notably, these three categories exhibited distinct ecological signatures. The most striking finding is the paradox between redundancy and insurance: fragile FRCs exhibited higher mean functional similarity (0.453) than robust FRCs (0.343), yet their dramatically lower response diversity (0.203 vs. 0.712) resulted in FII three times lower (0.078 ± 0.008 vs. 0.244 ± 0.006)—directly falsifying the assumption that more redundancy equals more stability.

Habitat-level comparisons revealed a pronounced spatial gradient in functional insurance (Fig. S8). Stream systems exhibited the highest mean FII (0.228 ± 0.009) and robust fraction (55.7% ± 6.4%), followed by reservoir samples (FII = 0.206 ± 0.009, robust fraction = 55.0% ± 9.2%), with no significant difference between these two natural habitat types (*P* > .05). In contrast, sewage samples showed significantly lower FII (0.146 ± 0.006, *P* < .001 vs. streams) and robust fraction (21.2% ± 4.0%, *P* < .001 vs. streams). The WTP_Z_ (FII = 0.147 ± 0.020, robust fraction = 30.7% ± 10.2%) and WTP_M_ (FII = 0.126 ± 0.020, robust fraction = 21.7% ± 7.4%) exhibited the lowest functional insurance, though neither differed significantly from sewage networks (*P* > .05). When aggregated into natural (stream, reservoir) versus engineered (sewage, WTP_Z_, WTP_M_) habitats, the disparity was highly significant (FII: 0.223 ± 0.008 vs. 0.142 ± 0.007, *P* < .0001). Engineered systems contained predominantly fragile FRCs (robust fraction accounts for only 23.5%), while natural environments harbored predominantly robust FRCs (55.5% ± 5.2%) (Fig. 4b, c). Generally, these results demonstrate that natural habitats maintain a substantially higher proportion of robustly insured functional redundancy, whereas engineered environments are dominated by fragile or non-redundant functional architectures. This reveals that aquatic ecosystem along a natural-engineered aquatic continuum all present an illusion of functional stability. From the perspective of conventional redundancy metrics, the functional redundancy of natural systems may have been underestimated; while engineered water systems (especially the sewage pipelines), which seem to be functionally stable, yet their redundant species respond synchronously to perturbations, offering no genuine insurance.

### Functional insurance, not redundancy, predicts carbon cycling efficiency and metabolic output stability

To examine whether the dual-layer functional insurance framework can be translated into measurable ecosystem-level consequences, we evaluated the relationships between metrics—effect layer, response layer, and the integrated FII—with CO_2_ and CH_4_ concentrations across all sampling sites. In aquatic ecosystems, elevated dissolved CO_2_ and CH_4_ typically reflect inefficient or incomplete carbon mineralization, particularly under conditions where organic matter is only partially oxidized [33, 34]. Thus, communities with higher FII often maintain more complete aerobic carbon turnover, thereby reducing the accumulation of these gaseous intermediates.

**Figure 5:**
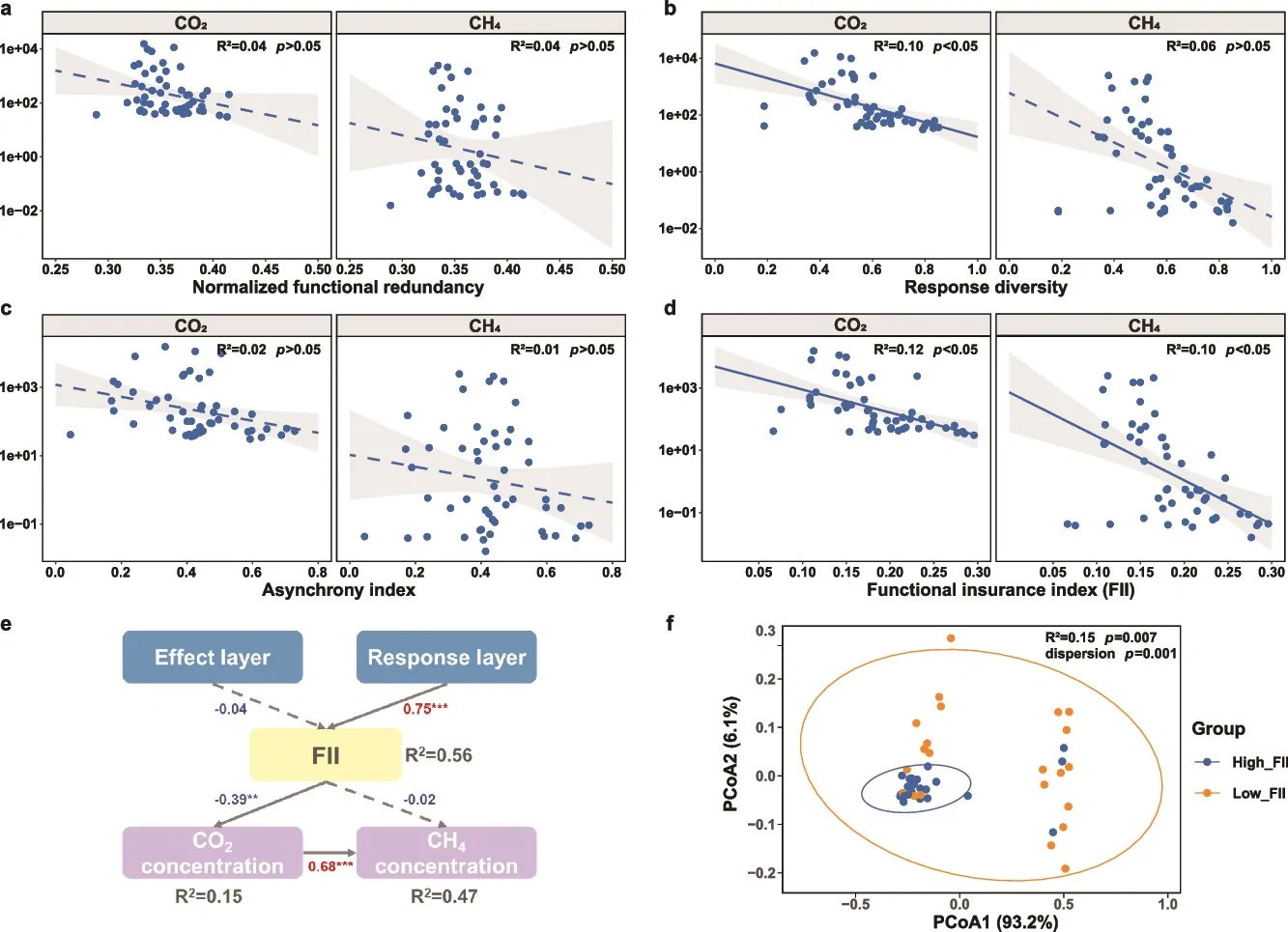
The relationships between the effect, response, and integrated layers and ecosystem functions (specifically CO_2_ and CH_4_ concentrations), as well as the association between the FII and DOM metabolite stability. (a-d) Linear relationship between normalized functional redundancy, response diversity, asynchrony index, FII, and CO_2_ and CH_4_ concentration. (e) Structural equation modeling (SEM) describes the direct and indirect effects of the effect layer, response layer, and FII on carbon dynamics (CO_2_, CH_4_ concentration). Results of the model fitting: *P* = .225, Fisher’s C = 10.608. The number adjacent to the arrow represents the effect size of the relationship. The red and blue numbers indicate positive and negative relationships, respectively; solid lines indicate significant relationships; dashed lines indicate non-significant relationships (*P* > .05); adjusted *P*-values are indicated by asterisks: ^*^P < .05, ^**^P < .01, ^***^P < .001. (f) PCoA of DOM compositional dissimilarity between high-FII and low-FII groups, categorized based on the median FII (0.366).

Linear regression analysis revealed a clear hierarchy in predictive power (Fig. 5a–d). Functional redundancy derived solely from genomic data showed no significant correlation with either CO_2_ or CH_4_ concentrations, indicating that the mere genomic potential for functional overlap does not directly reflect carbon dynamics. Response diversity was significantly correlated with CO_2_ concentration (*P* < .05) but not with CH_4_. The asynchrony index exhibited no significant relationship with either (*P* > .05 for both). In contrast, the FII was significantly correlated with both CO_2_ and CH_4_ concentrations, probably suggesting that only the composite metric—simultaneously capturing functional overlap (effect layer) and diversified environmental responses (response layer)—provides a reliable linkage to carbon dynamics.

SEM indicated that although the FII did not directly drive CH_4_ concentration; rather, it significantly regulated CO_2_ concentration (*P* = .005, Fig. 5e), which in turn exerted a strong positive cascading effect on CH_4_ production (*P* < .001), revealing an indirect ecological pathway driven by functional groups. These results showed that relying solely on either genomic or transcriptomic dimensions is insufficient. Instead, integrating them via the FII enabled us to capture the emergent properties governing ecosystem functioning.

To further validate the ecological implications of functional insurance at the metabolite level, we analyzed DOM composition using high-resolution mass spectrometry data. PCoA based on Bray–Curtis dissimilarity revealed that the first two axes explained 93.2% and 6.1% of the total DOM compositional variation, respectively (Fig. 5f). PERMANOVA (adonis) confirmed that DOM composition differed significantly between high-FII and low-FII groups (*P* = .007). Critically, these two groups exhibited strikingly different patterns of compositional variability. High-FII samples clustered tightly, displaying high compositional similarity and low within-group heterogeneity. Conversely, low-FII samples exhibited a substantially larger confidence ellipse. PERMDISP analysis confirmed that this disparity in within-group dispersion was statistically significant (*P* = .001), with low-FII groups exhibiting significantly greater beta-diversity than high-FII groups. This pattern directly supports the hypothesis: communities with high functional insurance can maintain more stable DOM profiles across spatially heterogeneous environments, whereas communities lacking functional insurance possess highly variable metabolite, reflecting greater stochasticity or vulnerability to local environmental perturbations. However, further examination of the two sub-clusters within low-FII samples along PC1 revealed a clear environmental association (Fig. S9a). Sub-cluster 1 predominantly comprised sewer network samples (raw municipal wastewater), whereas Sub-cluster 2 consisted of WTP samples together with several natural water samples (sites S6 and S7 in both May and Jan)—both of which are located downstream of the WTP discharge point, including the main receiving waterbody and its adjacent downstream areas. This grouping pattern suggests that treated effluent exerts a homogenizing effect on the DOM composition of downstream natural waters, effectively overprinting organic matter signatures across these otherwise distinct habitats. Although the two low-FII sub-clusters were compositionally distinct from the high-FII group (PERMANOVA, *P* < .001, Fig. S9a), their multivariate dispersions did not differ significantly (PERMDISP, *P* > .05). However, when all low-FII samples were pooled, their dispersion remained significantly higher than that of high-FII samples, also supporting the view that low insurance drives metabolic instability. To further verify the robustness of this finding, we also analyzed after excluding the three high-FII outlier samples that were visually separated from the main high-FII cluster in Fig. 5f. Results showed that the high-FII group continued to exhibit significantly lower compositional dispersion than the low-FII group (PERMANOVA: *R*^2^ = 0.29, *P* = .0002; PERMDISP: *P* = .001; Fig. S9b), confirming that the observed pattern was not affected by these outliers.

Taken together, the significant FII–CO_2_/CH_4_ correlations and the DOM compositional analysis provide converging lines of evidence that functional insurance, as quantified by FII, is not merely a theoretical construct but a mechanistically relevant predictor of ecosystem functioning. The dual-layer framework successfully bridges microbial community structure with ecosystem-level biogeochemical outcomes, linking the evidence chain from genomic redundancy through transcriptional insurance to metabolic stability.

## Discussion

### Functional redundancy across the aquatic continuum—but does it ensure stability?

Our analysis of the pooled MAGs indicates that high functional redundancy is a characteristic of the metacommunity spanning the natural-engineered aquatic continuum. Almost every recovered genome shares statistically significant functional overlap with multiple other community members. This finding aligns with previous observations that microbial communities harbor extensive functional redundancy [8, 9]. The dominance of Pseudomonadota, Bacteroidota, and Bacillota in hub positions suggests that these phyla act as functional generalists—a pattern consistent with their well-documented metabolic versatility in aquatic environments [35].

However, the translation of this universal genomic blueprint into sample-specific functional redundancy through personalized FRNs uncovered dramatic spatial heterogeneity. Sewage samples exhibited the highest TFR and modularity, reflecting the resource-rich, high-organic-load conditions that sustain large, functionally overlapping communities. In contrast, stream and reservoir systems showed significantly lower redundancy but maintained comparable normalized functional redundancy, suggesting that the per capita propensity of co-existing species to share functional capacity is a conserved ecological trait, regardless of total community size [9, 36]. This observation provides new evidence for the hypothesis that functional redundancy tends to scale proportionally with species richness [37], extending this principle from macro-ecological systems to environmental microbiomes. However, the mere presence of extensive functional overlap does not, by itself, guarantee functional stability. The critical question is whether these genomically redundant species can compensate for one another under environmental stress—a question that requires examining not just what species can do (effect trait), but how they actually respond (response trait) [14, 38].

### Anthropogenic management erodes response diversity and creates the illusion of stability

A central finding of this study is the decoupling of genomic functional redundancy from its ecological insurance value. While functional redundancy is ubiquitous, the transcriptional response heterogeneity within FRCs—the critical prerequisite for the insurance effect—varies dramatically across habitats. The strong positive correlation between response diversity and asynchrony index confirms the theoretical prediction of Yachi and Loreau [14]: when functionally similar species respond differently to environmental fluctuations, the resulting compensatory dynamics stabilize aggregate ecosystem functioning. Stream communities exhibited the highest response diversity and asynchrony index, consistent with the ecological reasoning that natural habitats, subject to stochastic and multidimensional environmental variation (e.g. flow, temperature, nutrient pulses), select for or maintain species with diversified environmental response strategies [12, 17]. Conversely, the progressive decline in response diversity along the natural-to-engineered gradient—from streams to reservoirs to sewer networks to municipal WTP_Z_ to industrial WTP_M_ systems—reveals a systematic erosion of response diversity under increasing anthropogenic management intensity. The most striking case was the sewage samples harbored the highest TFR, where a community yet the response diversity is not the highest. Such communities embody the illusion of functional stability: they appear functionally secure by conventional redundancy metrics, yet their synchronized response dynamics mean that all members fail simultaneously under stress—rendering their redundancy ecologically meaningless [11, 17]. This finding extends the concept of biotic homogenization [39, 40] from taxonomic and phylogenetic dimensions to a previously unrecognized functional response dimension, revealing that anthropogenic water management does not merely reduce diversity but fundamentally undermines the insurance mechanism that diversity is supposed to provide.

The ecological consequences of transcriptional homogenization in wastewater treatment systems are directly relevant to process stability and long-term performance. Although high redundancy may support efficient pollutant removal under stable operating conditions, homogenized transcriptional responses can reduce the insurance effect because functionally redundant taxa tend to respond synchronously rather than compensatorily when exposed to disturbances [14, 41]. Consequently, routine operational fluctuations, such as pH shifts, temperature changes, or transient toxic inputs, may lead to simultaneous downregulation of key metabolic functions and disproportionate functional losses [42]. This vulnerability may be further amplified under extreme environmental events (e.g. intense rainfall, episodic industrial discharges, etc.), because transcriptionally homogenized communities occupy a narrower response envelope and therefore have limited capacity to buffer stress across different environmental axes [12]. From a long-term perspective, such reduced response diversity may also weaken the ability of engineered microbial communities to accommodate gradual changes in influent composition, emerging contaminants, warming conditions, and stricter treatment requirements. These findings suggest that maintaining high microbial diversity and functional redundancy alone may not be sufficient to ensure stable wastewater treatment performance. Instead, management strategies that promote response diversity among redundant taxa may help enhance the functional insurance of engineered systems.

### The functional insurance index reveals the redundancy–insurance decoupling

FII represents the first quantitative implementation of the biodiversity-insurance hypothesis [14] in microbial ecology. While previous studies have separately quantified functional redundancy through genomic similarity [43] or reported response diversity through species-level turnover [11], no framework has explicitly integrated these two dimensions into a single, mechanistically interpretable index.

Our dual-layer framework advances the biodiversity–stability theory in three respects. First, whereas the classical insurance hypothesis [14] and its extensions [15] were formulated and tested primarily through species biomass fluctuations, our framework operationalizes insurance specifically for microbial communities by leveraging multi-omics data that capture both genomic potential (metagenomics) and realized activity (metatranscriptomics). Second, whereas previous applications of response diversity to microbial systems have relied on phylogenetic distance or trait-based proxies [17], our approach directly quantifies transcriptional response heterogeneity—the actual divergence in taxa expression under environmental variation—providing a more functionally explicit measure of response diversity. Third, whereas recent studies of microbial functional redundancy have focused on cataloging genomic overlap without assessing its insurance value [9, 18], our FII explicitly integrates effect similarity with response diversity into a single metric, enabling the critical distinction between “redundancy that insures” and “redundancy that merely duplicates.” This integration closes a persistent gap between biodiversity–stability theory and its application to the microbial world.

The ecological validity of the three-category classification was confirmed by a revealing phenomenon. Fragile FRCs exhibited higher mean functional similarity than Robust FRCs, yet their dramatically lower response diversity resulted in FII being substantially lower. This directly falsifies the intuitive assumption that more redundancy equals more stability—the very assumption that underlies conventional assessments of microbial community health in engineered water systems. Functionally identical species that respond identically to stress are merely synchronized backups: they create an illusion of insurance without its substance. Additionally, unlike single-metric approaches (e.g. Shannon diversity), the categorization of FII into three distinct classes provides an actionable basis for ecological diagnostics. For example, robust FRCs can emerge as a diagnostic metric capable of distinguishing genuinely resilient from superficially redundant communities. Specifically, fragile FRCs identify potential tipping points where communities may appear healthy but lack genuine resilience, representing targets for monitoring or management intervention. At the habitat level, the significantly higher robust fraction in natural environments compared to engineered systems carries an important implication. The water treatment systems that humanity depends upon for pollution control may be far more ecologically fragile than their microbial diversity indicators suggest. Conventional assessments based on species richness or TFR systematically misestimate the functional stability of these systems because they conflate the capacity for functional substitution with the realized likelihood of compensatory response. The FII framework pierces this illusion by distinguishing between superficially healthy (high redundancy, low insurance) and genuinely resilient (high insurance) communities.

The proposed FII extends existing stability metrics by asking not only how much redundancy exists, but also whether such redundancy is likely to provide realized functional insurance under environmental change. By integrating effect trait (genomic functional similarity) and response trait (transcriptional response heterogeneity), FII provides a measure of whether redundant taxa can actually buffer functional performance when conditions change. Although developed here for aquatic ecosystems, the framework has the potential to be applied to other ecosystems where paired metagenomic and metatranscriptomic data are available, such as soils, marine microbial communities, and host-associated microbiomes. Moreover, our findings may help explain several puzzling observations from other ecosystems and published microbiome datasets. In activated sludge systems, sudden and unexplained performance failures despite stable microbial diversity have been repeatedly documented [44, 45]—a pattern consistent with high redundancy, low insurance communities that appear stable until a perturbation exceeds their narrow shared response envelope. In soil ecosystems, microbial community composition often shows limited resilience despite high functional redundancy [8, 11]. A paradox that the redundancy–insurance decoupling could resolve: if soil microbial redundancy is similarly fragile (i.e. lacking response diversity), then compositional shifts would directly compromise function rather than being buffered. It can be considered that FII may offer both a management diagnostic for different microbial communities and an early-warning signal of ecosystem vulnerability that complements classical statistical indicators.

### The consequence of the illusion: only insurance, not redundancy, predicts ecosystem functioning

The consequence of the redundancy–insurance decoupling is stark. FII—but not functional redundancy, response diversity, or asynchrony alone—significantly predicted CO_2_ and CH_4_ concentrations and DOM compositional stability, providing direct evidence that the illusion identified above has real ecosystem-level consequences. However, the negative correlations between FII and CO_2_/CH_4_ should be interpreted with caution: they do not imply that higher insurance weakens ecosystem function. Rather, in the context of aquatic ecosystems, supersaturation of these gases is a hallmark of disrupted or inefficient carbon processing [46]. Communities with robust functional insurance maintain metabolic continuity under fluctuating conditions, favoring oxidation of organic matter and minimizing the accumulation of partially reduced end-products. This interpretation aligns with the broader ecological theory that functional insurance primarily confers resistance against functional degradation, and our DOM stability analysis directly supports this by showing that high-FII communities can maintain more stable compositional profiles. These findings provide compelling evidence that the integration of effect layer and response layer reveals emergent properties unattainable by individual metrics. This result has both theoretical and practical significance.

Theoretically, it supports the hypothesis that ecosystem functioning depends not on redundancy per se, but on the interaction between functional overlap and response heterogeneity [14, 15, 47]. The failure of genomic-level redundancy to predict carbon dynamics underscores a fundamental limitation of metagenomics: gene presence does not guarantee expression. Research findings on response diversity suggest that transcriptomic heterogeneity can only reflect partial aspects of carbon dynamics, which may indicate that this approach also has certain limitations. Practically, the FII–CO_2_/CH_4_ relationship suggests that functional insurance could serve as a bioindicator for carbon dynamics potential in aquatic ecosystems. The multi-level functional analysis of carbon-metabolism genes provides a mechanistic interpretation of how functional insurance regulates carbon-cycle processes (Fig. S10). The taxonomic contribution patterns revealed that while *Methanothrix* and *Megamonas* play an important role in carbon metabolism in low-FII groups, high-FII groups are characterized by greater relative contributions from aerobic organism (such as aerobic methanotrophs: *Methylomonas* and *Methylobacter*), suggesting a systematic shift from anaerobic to aerobic carbon processing pathways as insurance increases. At the transcriptional level, the enrichment of carbon fixation and organic matter degradation pathways among differentially expressed genes—coupled with the constitutive expression of core housekeeping functions (e.g. central carbon metabolism) regardless of FII status—indicates that insurance does not operate through the presence or absence of metabolic genes, but rather through the transcriptional heterogeneity to modulate pathway usage in response to environmental variation. Communities with low-FII possess the same basal metabolic toolkit as high-FII communities, yet they lack the regulatory capacity to regulate alternative carbon-processing pathways when conditions change. This pattern aligns with the response diversity concept [12]: insurance arises not from functionally redundant taxa, but from the diversity of transcriptional heterogeneity among these taxa. Thus, these findings bridge community ecology and biogeochemical functioning. Given the growing concern over greenhouse gas emissions from urban water infrastructure [48], a metric that integrates community-level functional insurance with ecosystem-level carbon dynamics could aid in monitoring and managing.

The DOM compositional analysis provides an independent, metabolite-level validation of the functional insurance mechanism [49]. The significantly tighter clustering of DOM profiles in high-FII samples demonstrates that communities with high functional insurance possess more predictable and stable metabolites. This is the definition of the insurance effect at the metabolite level: when functionally redundant species asynchronously compensate for one another’s transcriptional fluctuations, the net metabolic output remains buffered against environmental perturbation. The markedly higher DOM compositional heterogeneity in low-FII samples mirrors the stochastic from communities lacking compensatory response dynamics. This phenomenon indicates that variations in microbial communities are influenced by the composition and availability of resources in their surrounding environment [50]. Additionally, the convergence of WTP and downstream natural samples within a single low-FII sub-cluster further implies that anthropogenic discharge can override habitat-specific DOM signatures. When all low-FII samples were considered together (without environmental partitioning), they exhibited significantly greater dispersion than the high-FII group. Taken together, these results suggest that the stabilizing effect of high functional insurance is most clearly detected at the overall community variance level, while environmental heterogeneity contributes to compositional separation.

## Conclusion

Overall, our research closes the evidence chain from genotype to phenotype to chemotype: genomic redundancy—transcriptional response heterogeneity—metabolic stability. These findings expose an illusion of functional stability in a natural-to-engineered aquatic continuum and challenge the prevailing practice of equating functional redundancy with functional insurance. More broadly, our findings refine current interpretations of microbial ecosystem resilience under environmental change. The decoupling between functional redundancy and insurance challenges the common assumption that high microbial diversity or redundancy necessarily translates into stability [8, 9]. This distinction is important for biodiversity conservation and ecosystem management because it suggests that maintaining functional stability requires preserving response diversity, not merely species richness or redundant functional capacity. In managed ecosystems increasingly exposed to global change, such as agricultural soils, engineered water systems, and aquaculture environments, environmental homogenization may erode this response diversity while leaving apparent redundancy intact. Therefore, the FII framework, which identifies the hidden gap between them under disturbances, highlights why functional insurance assessments—using effect and response traits—should be standard in microbial community health diagnostics.

The dual-layer framework established here provides the conceptual foundation and analytical toolkit to pierce this illusion, offering comprehensive evidence linking the insurance hypothesis to real-world ecosystem functioning. As the framework is extended to incorporate temporal dynamics, additional omics layers, and cross-system validation, we anticipate that the redundancy–insurance decoupling documented here will prove to be a general pattern. This will have a profound impact on our assessment, monitoring, and management of the ecological resilience of global water systems.

## Supplementary Material

Supplemental_Material-revision3_ycag229

## Data Availability

All datasets generated in this study are publicly available in the National Genomics Data Center (NGDC) repository. Sequence data are deposited under BioProject accession numbers PRJCA037163 and PRJCA037164 for metagenome and metatranscriptome, respectively.
