# Supplementary material for: The illusion of functional stability: functional redundancy does not equal functional insurance in a natural-engineered aquatic continuum
Source: ISME Commun. 2026 Aug 24;6(1):ycag229. doi: 10.1093/ismeco/ycag229 (PMC13600385; doi:10.1093/ismeco/ycag229)
Supplement: Supplementary file 1 [file supplemental_material-revision3_ycag229.docx]

**Title:** The illusion of functional stability: functional redundancy does not equal functional insurance in a natural-engineered aquatic continuum

**Running title:** Redundancy is not insurance

**Authors:** Qiong Tang^1,2^, Zhe Li^1,2*^, Lunhui Lu^1,2^

1 State Key Laboratory of Lake and Watershed Science for Water Security, Chongqing Institute of Green and Intelligent Technology, Chinese Academy of Sciences, Chongqing 400714, China.

2 Chongqing School, University of Chinese Academy of Sciences, Chongqing 400714, China.

^*^ Corresponding author: [lizhe@cigit.ac.cn](mailto:lizhe@cigit.ac.cn)

Corresponding author’s mailing address: Zhe Li, State Key Laboratory of Lake and Watershed Science for Water Security, Chongqing Institute of Green and Intelligent Technology, Chinese Academy of Sciences, No. 266 Fangzheng Avenue, Liangjiang New Area, Chongqing 400714, China.

# Supplementary Methods

## Supplementary Method 1

**Dissolved gas and DOM analysis**

Dissolved CH_4_ and CO_2_ concentrations in the water phase at each sampling site were detected with the headspace approach by a G2201-*i* Isotopic Analyzer (Picarro®, USA).

Dissolved organic matter (DOM) composition was characterized by high-resolution mass spectrometry analysis (mass range: 100-1000 Da; elemental constraints: C_1-50_, H_1-125_, O_1-60_, N_0-3_, S_0-2_, and P_0-2_). Molecular formulas were assigned via the TRFU algorithm. They were categorized into ten groups: lipid-like, protein-like, amino sugar-like, carbohydrate-like, aliphatic-like, condensed aromatic-like, polyphenol-like, highly unsaturated and phenolic-like, lignin-like, and tannin-like [1]. DOM composition was represented as a sample × molecular formula intensity matrix for downstream multivariate analyses.

**DOM sample pre-processing**

Sample processing and testing protocols were detailed as follows. For sample collection, water samples were individually collected from each system and each set of incubation experiment conditions. These samples were subsequently filtered through a 0.7 μm GF/F glass fiber membrane (Whatman®, UK). Following filtration, 1 mL of 6 mol·L^-1^ hydrochloric acid was added to the filtrate to adjust its pH to 2, ensuring the stabilization of the sample properties. The treated samples were then stored in the dark at 4°C for further processing, with a holding time of 24 hours.

Solid-phase extraction (SPE) was performed using an Agilent Bond Elut PPL column packed with 500 mg of polystyrene-divinylbenzene copolymer (column volume: 6 mL). The procedure began with the sequential activation and rinsing of the column using 18 mL of chromatography-grade methanol, followed by 18 mL of acidified ultrapure water (pH = 2). The aqueous sample, acidified to pH = 2, was then passed through the PPL column at a flow rate of less than 5 mL/min. The column was subsequently rinsed with 18 mL of acidified ultrapure water (pH = 2) to remove inorganic salts and then dried using nitrogen gas. Finally, the organic compounds were eluted from the column with 8 mL of methanol and the eluate was adjusted to a final volume of 10 mL with methanol. The extracts were collected in a brown reagent bottle and stored in the refrigerator (-20°C) until further analysis.

The Orbitrap-MS analysis was conducted to determine the molecular composition of dissolved organic matter (DOM) using an Orbitrap mass spectrometer in negative ion electrospray mode (Thermo Eclipse model). The DOM sample was automatically injected via a syringe pump at a flow rate of 20 μL/min. The experimental settings included a spray voltage of -3.8 kV, sheath gas pressure of 5 arb, and auxiliary gas pressure of 2 arb. The mass spectrometer operated at a resolution of 1,000,000 FWHM, with a mass acquisition range of 100 to 1,000 m/z and an acquisition time of 7 minutes. Before analysis, external calibration was performed using a standard mixture (Thermo Pierce™ FlexMix™ calibration solution). Following data acquisition, internal calibration was carried out using compounds commonly present in DOM.

## Supplementary Method 2

**Physicochemical properties and environmental differentiation**

Water temperature, pH, and dissolved oxygen (DO) were measured *in situ* with YSI® EXO2 (YSI Incorporated, Ohio, USA). Dissolved organic carbon (DOC) were quantified via TOC analyzer (Vario® TOC, Elementar, Germany). Total nitrogen (TN), nitrate nitrogen (NO_3_-N) and total phosphorus (TP) were analyzed with a UV spectrophotometer according to standard methods.

Hierarchical clustering based on Bray-Curtis dissimilarity of both microbiological and physicochemical characteristics across the five systems (i.e., reservoir, stream, sewage, WTP_Z_, and WTP_M_) revealed that microbial communities with similar taxonomic compositions occupy comparable environmental niches, thereby confirming the reliability of our sampling strategy and the robustness of the obtained datasets (Fig. S2).

## Supplementary Method 3

**Rationale for using a bimodal threshold to classify Functional Redundancy Clusters (FRCs) into ecological types**

Functional similarity (0.30) was set substantially above the permutation null model baseline (0.2456) (Fig. S3), ensuring that only FRCs with statistically robust genomic overlap were considered functionally redundant. The threshold for response diversity corresponds to a mean intra-cluster correlation of 0.50, representing the boundary between moderately synchronous (< 0.5, fragile) and asynchronous/independent (≥ 0.5, insured) responses, consistent with the theoretical prerequisites of the insurance hypothesis [2]. Grid-based sensitivity analysis across multiple threshold combinations (functional similarity: 0.25-0.35; response diversity: 0.40-0.60) confirmed that the (0.30, 0.50) combination yielded the most ecologically balanced three-way partition (Robust: 35.4%, Fragile: 29.6%, Non-redundant: 31.3%).

# Supplementary Figures

**Fig. S1** The location of sampling sites in the Yanjin, Chishui River basin.

**Fig. S2** Relationship between microbial community structure and physicochemical factors. **A** The microbial community structures and the corresponding site’s physicochemical data are expressed as Bray-Curtis dissimilarity plotted in a dendrogram. Identical samples in a tree diagram are connected by a line and colored according to the group they belong to. Reservoir: (R1~R3), stream: (S1~S10), sewage: (P1~P7), WTP_Z_: (W_Zi_, W_Za_, W_Zo_) and WTP_M_: (W_Mi_, W_Ma_, W_Mo_). **B** Non-metric multidimensional scaling (NMDS) diagram illustrates the microbial communities structure among different systems.

**Fig. S3** Permutation-based null model for determining the significance cutoff of functional similarity. The significance threshold (0.2456) is defined by the 95th percentile of the null distribution. The vertical axis represents the number of species pairs.

**Fig. S4** **a** Visualization of Genomic Content Network (based on 443 MAGs). Nodes are colored by Phylum; edges represent Weighted Jaccard Similarity. **b** Rank abundance distribution (top 30) of microbial communities across different taxonomic levels.

**Fig. S5** Boxplot of total nodes (**a**), total functional redundancy (**b**) and normalized functional redundancy (**c**) among reservoir, stream, sewage, WTP_Z_, and WTP_M_ systems; Comparison of the total functional redundancy between January and May (**d**); Lowercase letters above the boxes represent the results of statistical significance test (*p* < 0.05, ANOVA with post-hoc Tukey HSD Test) for each group. Groups with different letters indicate significant differences between groups, and groups with the same letter means non-significant differences between groups.

**Fig. S6** The relationship between modularity and total functional redundancy, network density.

**Fig. S7** Schematic illustration of genuine functional insurance versus illusion of insurance in functionally redundant communities. **a** Genuine Insurance: Species exhibit response diversity (multiple colors). Upon perturbation, while some species decline, others compensate, thereby maintaining the overall ecosystem function. **b** Illusion of Insurance: Species exhibit homogenized responses (a single color). Upon perturbation, all species decline simultaneously, preventing any compensation and leading to the collapse of the aggregate function.

**Fig. S8** The changes of functional insurance index (FII) and robust redundancy fraction in each sample in May (M) and January (J).

**Fig. S9 a** Principal coordinate analysis (PCoA) of DOM composition comparing High-FII and Low-FII subclusters. PCoA based on Bray-Curtis dissimilarity showing the separation of DOM compositional profiles among High-FII samples and two Low-FII subclusters (Low-FII_Sub1 and Low-FII_Sub2) divided along PC1. Sub-cluster 1 predominantly comprises sewer network samples, while Sub-cluster 2 comprises WWTP samples and downstream natural water samples (sites S6 and S7 in May and Jan). **b** PCoA of DOM composition based on Bray-Curtis dissimilarity, showing High-FII and Low-FII samples after removal of the three High-FII outliers (sites P6 in May and Jan, and sites P7 in Jan) that diverged from the main High-FII cluster in Fig. 5f.

**Fig. S10 a** Species and functional contribution barplot. The plot shows the relative contribution of dominant genera to the carbon‑metabolism gene pool across all samples, with each colored segment representing the proportional contribution to a specific KEGG pathway (F01–F20). **b** Contribution heatmap comparing High‑FII and Low‑FII groups. The heatmap displays the relative contribution of dominant genera to carbon‑metabolism pathways in High‑FII and Low‑FII groups. **c** Volcano plot of differential expression analysis. Differential expression analysis of carbon‑metabolism genes between High‑FII and Low‑FII groups. Red and blue points represent significantly upregulated (n = 4,240) and downregulated (n = 4,094) genes, respectively (|log_2_FC| > 1, FDR < 0.05). Grey points indicate genes with no significant expression difference (n = 9,780). **d** KEGG enrichment bubble plot of differentially expressed genes. The plot shows significantly enriched KEGG pathways among the differentially expressed genes. The size of each bubble represents the number of genes annotated to the corresponding pathway, and the color gradient indicates the adjusted *p*‑value (log_10_P_adjust).

# Reference

1.	Ma K, Li YY, Song W, Zhou JY, Liu X, Wang MQ *et al*: Disentangling drivers of mudflat intertidal DOM chemodiversity using ecological models. *Nat Commun* 2024, 15(1):13.

2.	Yachi S, Loreau M: Biodiversity and ecosystem productivity in a fluctuating environment: The insurance hypothesis. *Proc Natl Acad Sci U S A* 1999, 96(4):1463-1468.
